# Intervention effects on children’s movement behaviour accumulation as a result of the *Transform-Us!* school- and home-based cluster randomised controlled trial

**DOI:** 10.1186/s12966-022-01314-z

**Published:** 2022-07-07

**Authors:** Simone J. J. M. Verswijveren, Nicola D. Ridgers, Josep A. Martín-Fernández, Sebastien Chastin, Ester Cerin, Mai J. M. Chinapaw, Lauren Arundell, David W. Dunstan, Clare Hume, Helen Brown, Jacqueline Della Gatta, Jo Salmon

**Affiliations:** 1 Institute for Physical Activity and Nutrition (IPAN), Deakin University, Geelong, Australia; 2 Alliance for Research in Exercise, Nutrition and Activity (ARENA), University of South Australia, Adelaide, South Australia Australia; 3 Department of Computer Science, Applied Mathematics and Statistics, University of Girona, Girona, Spain; 4 School of Health and Life Sciences, Glasgow Caledonian University, Glasgow, UK; 5 Mary McKillop Institute for Health Research, Australian Catholic University, Melbourne, Victoria Australia; 6 Department of Public and Occupational Health, Amsterdam UMC, Vrije Universiteit Amsterdam, Amsterdam Public Health Research Institute, Amsterdam, the Netherlands; 7 Baker Heart and Diabetes Institute, Melbourne, Victoria Australia; 8 School of Public Health, University of Adelaide, Adelaide, South Australia Australia

## Abstract

**Background:**

It is unknown if and how children’s movement behaviour accumulation patterns change as a result of physical activity and/or sedentary behaviour interventions. It is important to establish the effectiveness of interventions targeting changes in such accumulation patterns. This study aimed to investigate the effect of the *Transform-Us!* school- and home-based intervention program on children’s movement behaviour accumulation patterns, focusing on sporadic accumulation versus time in bouts.

**Methods:**

Baseline and post-intervention (18 months) accelerometer data from the *Transform-Us!* 2 × 2 factorial design cluster randomised controlled trial was used (Melbourne, 2010–2012; analytical sample *n* = 267; aged 8–9 years). Linear mixed models were fitted to examine effects of three different interventions (targeting increases in physical activity [PA-I], reductions in sedentary time [SB-I], or both [PA + SB-I]) compared to a usual practice (control) group on post-intervention movement behaviour accumulation compositions with eight components, including sporadic time and bouts of sedentary time, and light-, moderate- and vigorous-intensity physical activity.

**Results:**

Intervention effects on distribution of time in the post-intervention waking movement behaviour accumulation composition (adjusted for baseline composition) were small and not significant. However, visual inspection of the change in compositions over time revealed that only groups with a sedentary behaviour intervention component (SB-I and PA + SB-I) reduced time in sedentary bouts, compared to the overall sample compositional mean. In addition, the SB-I group was the only group with an increase in vigorous-intensity physical activity. The combined intervention group (PA + SB-I) was characterized by the largest proportional increase in MPA bouts. The usual practice group was characterized by the largest proportional increases in both sporadic and bouts of sedentary time.

**Conclusions:**

This study showed some early evidence to suggest that the “break up your sitting” message may result in greater impact than the “move more” message. Future research, including larger sample sizes, should investigate if this type of messaging is indeed more effective in changing movement behaviours and ultimately child health.

**Trial registration:**

International Standard Randomized Controlled Trial Number ISRCTN83725066; Australian New Zealand Clinical Trials Registry Number ACTRN12609000715279.

**Supplementary Information:**

The online version contains supplementary material available at 10.1186/s12966-022-01314-z.

## Background

The Australian 24-h movement guidelines state that children (5–17 years) should engage in ≥60 minutes of moderate- to- vigorous- (MVPA) and several hours of light-intensity (LPA) physical activity per day for optimal health [1]. In addition, it is recommended that children limit sedentary recreational screen time to no more than 2 h per day and break up long periods of sitting as often as possible [1]. Whilst these guidelines acknowledge the collective importance of these waking movement behaviours for child health, no specific guidance on how these should be combined or accumulated is available. For example, the guidelines do not explain whether the ≥60 minutes of MVPA per day are best accumulated sporadically across the day or in sustained periods (i.e., prolonged bouts of movement).

Some emerging evidence suggests that how movement behaviours are accumulated may be related to health markers, such as adiposity and fitness, in children (e.g. [2–5],). Consequently, some children’s movement behaviour interventions (e.g. [6],) have been developed to reduce their sedentary time and promote physical activity by breaking up prolonged periods of sitting, as well as other strategies that may impact on accumulation patterns. However, the existing intervention evidence appears limited to studies focusing on changing total durations of specific movement behaviours (e.g., increasing overall MVPA or decreasing sedentary time) [7, 8]. There is currently a poor understanding of how interventions can change overall movement accumulation patterns and systematic reviews have highlighted this gap in the literature [7, 8]. Intervention study data should be analysed with a focus on the patterns of multiple movement behaviours across the activity spectrum to explore whether these are modifiable through intervention strategies. For such analyses, approaches that appropriately deal with co-dependency between behaviours (e.g., compositional data analysis [CoDA] [9]) are required. To date, no studies have used such statistical approaches in children to assess intervention effects on accumulation patterns, and specifically sporadic behaviours versus bouts. This information is critical for informing the design, delivery and evaluation of interventions to change accumulation patterns, which may have the potential to benefit health.

*Transform-Us!* ([10]; Salmon J, Arundell L, Cerin E, Ridgers ND, Hesketh KD, Daly RM, et al: The Transform-Us! cluster RCT: 18- and 30-month effects on children’s physical activity, sedentary time and cardiometabolic risk markers, unpublished) was a school- and home-based intervention that aimed to increase primary school children’s physical activity and decrease their sedentary time. A range of strategies were used, including intervention components that focused on accumulation patterns, such as breaking up sitting using an active curriculum (e.g., ‘active mathematics’) and offering ‘activity breaks’ during class time ([10], Salmon J, Arundell L, Cerin E, Ridgers ND, Hesketh KD, Daly RM, et al: The Transform-Us! cluster RCT: 18- and 30-month effects on children’s physical activity, sedentary time and cardiometabolic risk markers, unpublished). The intervention was effective in lowering the total daily duration of children’s sedentary time (by 27 min at 18-months and 33 min at 30-months compared to usual practice) and increasing daily MVPA (by 5 minutes at 18-months) (Salmon J, Arundell L, Cerin E, Ridgers ND, Hesketh KD, Daly RM, et al: The Transform-Us! cluster RCT: 18- and 30-month effects on children’s physical activity, sedentary time and cardiometabolic risk markers, unpublished). However, if and how children’s movement behaviour accumulation patterns changed during the intervention has not yet been examined. This is important for gaining insights into the implementation of strategies targeting changes in activity patterns (e.g., breaking up sitting every 30 minutes with a 2–3 minute bout of activity) and understanding what accumulation patterns could be targeted in interventions to benefit health. Therefore, the aim of this study was to investigate the effect of the *Transform-Us!* school- and home-based intervention program on children’s movement behaviour accumulation patterns, focusing on sporadic accumulation versus time in bouts.

## Materials and methods

### Study design

The *Transform-Us!* 2 × 2 factorial design cluster randomised controlled trial including three different interventions (targeting increases in physical activity, reductions in sedentary time, or both) and a usual practice control group was delivered in Melbourne, Victoria, Australia between Feb-Jun 2010 and Nov-Dec 2012. Study details can be found in the previously published study protocol [10] and outcomes papers ([11, 12], Salmon J, Arundell L, Cerin E, Ridgers ND, Hesketh KD, Daly RM, et al: The Transform-Us! cluster RCT: 18- and 30-month effects on children’s physical activity, sedentary time and cardiometabolic risk markers, unpublished). The trial is registered with the International Standard Randomized Controlled Trial (ISRCTN83725066) and the Australian New Zealand Clinical Trials Registry (ACTRN12609000715279). Ethical approval was obtained by the Deakin University Human Research Ethics Committee (EC141–2009), the Victorian Department of Education and Early Childhood Development (2009–000344), and the Victorian Catholic Education Office (1545).

### Recruitment and randomization

Primary schools within 50 km of the Melbourne Central Business District with ≥300 students and at least 2 year 3 (aged 8–9 years) classes were eligible to receive an invitation for the study (*n* = 219 schools). Schools were identified as being in low, mid and high socioeconomic status (SES) areas based on the first, third and fifth quintile of the Australian Bureau of Statistics Socio-Economic Index for Areas [13]. Schools in low (*n* = 74), mid (*n* = 74) and high (*n* = 71) SES areas were then randomly ordered with probabilistic weighting according to the number of students enrolled [10, 14]. In total, 127 primary schools were approached of which 20 (low: 8; mid: 11; high: 1) agreed to participate and were randomly allocated to one of four groups: 1) usual practice control group (C); 2) intervention group targeting increases in physical activity (PA-I); 3) intervention group targeting reductions in sedentary time (SB-I); and 4) intervention group targeting both movement behaviours (PA + SB-I). Fig. S1 in Additional file 1 presents a flow diagram with recruitment and randomisation participant numbers. Detailed randomisation and masking procedures have been described previously [10, 15]. While all children in the intervention schools (PA-I, SB-I and PA + SB-I) received the program, only those with written parental consent were included in the evaluation. Parents could elect for their child to complete any combination of the behavioural, demographics and health assessments described below.

### Intervention

The intervention program was delivered by accredited Victorian Institute of Teaching classroom teachers and targeted physical activity and/or sedentary behaviours in the school and home settings [10]. The intervention used educational, pedagogical, behavioural, social and environmental strategies [10] and was based on cognitive theory [16], behavioural choice theory [17], and ecological systems theory [18]. In the first year of the intervention, all Year 3 teachers in the intervention groups were provided with detailed lesson plans and asked to deliver nine key learning messages, focusing on sedentary behaviour and/or physical activity depending on the intervention allocation (PA-I, SB-I or PA + SB-I). The intervention messages for students through the health lessons included for example ‘making active choices’, ‘importance of being active’, and ‘self-monitoring activity levels’. Homework tasks involved either adapting the child’s existing homework (e.g., to complete it while standing at a bench) or incorporating a component/task that involved reducing sedentary time or increasing physical activity (e.g., switch off screens and/or create an active game to perform with their parent). In the second year of the intervention, Year 4 teachers were asked to deliver an additional nine key learning messages which expanded/built on the previous year. Nine newsletters were sent to parents via the school each year (18 in total) that reinforced the sedentary behaviour and/or physical activity key messages (depending on the intervention group) and promoted family involvement (e.g., go for a walk with parents and count letterboxes in their street).

In addition to the key learning messages, the PA-I grade 3 classes were provided with pedometers, physical activity and novel circus equipment in classroom tubs (e.g., juggling balls), and the school was provided with asphalt line markings and signage promoting physical activity [10]. Teachers were asked to encourage and support children’s physical activity during recess and lunchtime [10]. In contrast, the SB-I teachers were asked to interrupt children’s seated class time once a day using a 30-minute active curriculum (e.g., ‘active mathematics’), to break up children’s sitting with a 2-minute ‘activity breaks’ approximately every 30 minutes, and to adapt homework tasks to break up sitting and incorporate standing [10].

The PA + SB-I schools were provided with all of the above physical activity and sedentary behaviour strategies. The usual practice control schools received the intervention information and supporting materials once the study was completed. Further intervention details have been previously described ([10–12], Salmon J, Arundell L, Cerin E, Ridgers ND, Hesketh KD, Daly RM, et al: The Transform-Us! cluster RCT: 18- and 30-month effects on children’s physical activity, sedentary time and cardiometabolic risk markers, unpublished).

### Data collection and measures

Data were collected by trained research staff who were blinded to intervention group allocation at baseline [10]. The present study used data from two timepoints, namely Feb-May 2010 (baseline) and Nov-Dec 2011 (18 months; post-intervention). Data procedures at these time-points were the same for all measures.

#### Accelerometry

Participants were provided with an ActiGraph GT3X (ActiGraph, Pensacola, FL, USA) accelerometer and asked to wear it on their right hip for eight consecutive waking days, except during water-based activities [10]. The normal frequency filter was selected and data were collected and stored in 15-second epochs [19]. These data were then downloaded using the ActiLife software (ActiGraph, Pensacola, FL, USA) and reduced to daily values using a customised Excel macro. Non-wear time was defined as ≥20 minutes of consecutive zeros [19] and valid days were set at a minimum of 8 hours on weekdays or 7 hours on weekend days [20]. To optimize the sample size, participants had to have ≥3 valid days at both time points to be considered for inclusion in the study. This, in combination with the minimum valid wear time per day, gives reasonable reliability and power for assessing children’s habitual movement behaviours [20].

To determine the total duration of time in LPA, moderate- (MPA), and vigorous-intensity (VPA) physical activity, the age-specific cut-points developed by Freedson and colleagues [21] were adapted to 15-s epoch cut-points. Sedentary time was defined as ≤25 counts per 15-s [19]. Time in each intensity was then divided into sporadic time versus uninterrupted bouts of a specific intensity, using ≥5 min for sedentary bouts, and ≥ 1 min for physical activity bouts (including LPA, MPA and VPA), respectively, based on previous work using this dataset [22]. No tolerance (i.e., interruption in intensity) was allowed in defining the bouts, based on previous recommendations for sedentary bouts [15] and in the absence of recommendations for physical activity bouts. Mean values for all accelerometry variables were calculated over all valid days.

#### Participant characteristics

Child age and sex were self-reported at baseline or, if missing, collected from parental proxy-reports. School SES was determined using the postal code in the national 2006 Socio-Economic Indexes for Areas Index of Relative Socioeconomic Advantage and Disadvantage of the Socioeconomic Indexes Areas and classified as low (i.e., first quintile), mid (i.e., third quintile), and high (i.e., fifth quintile) SES [13]. Height, weight, and waist circumference (WC) were measured at school by trained research staff using standardized procedures with portable stadiometers (SECA 220, Los Angeles, CA, USA) and digital scales (Wederburn Tanita, Melbourne, Vic, Australia) [10, 23]. Body Mass Index (kg/m^2^) was calculated and converted to age- and sex- standardized z-values (zBMI) using World Health Organization (WHO) child growth charts [24].

### Statistical analysis

Analyses were performed in STATA version 16 (STATACorp, College Station, TX, USA) and RStudio version 1.4.453 (R version 3.6.3; R Foundation for Statistical Computing, Vienna, Austria), using the compositions (*acomp* framework [23]), zCompositions (*multLN* zero replacement [14]) and lme4 (*lmer* function [25]) packages.

An analytical sample was created consisting of participants that had valid accelerometry at both timepoints (as defined above) and covariates (age, sex and SES) data at baseline. Participant characteristics (mean ± standard deviation [SD]) were compared using t-tests (continuous variables) and chi-square tests (categorical variables) between the analytical sample and those participants excluded for incomplete data. Baseline characteristics (mean ± SD) for the four groups (usual practice, PA-I, SB-I and PA + SB-I) were presented separately, but not compared using inferential tests as per the Consolidated Standards of Reporting Trials recommendations [26].

Accelerometry variables were checked for zeros. This was warranted as transforming values to isometric log ratio (*ilr)* pivot coordinates cannot be done for 0 as dividing by 0 or taking the logarithm of 0 are undefined mathematical operations [9]. Most accelerometry-based variables did not contain zeros, except for the VPA bouts component at both baseline (*n* = 1; < 1%) and post-intervention (*n* = 6; 2%). Consequently, these were replaced using the multiplicative lognormal imputation (*multLN*) replacement method of the zCompositions package with the observed minimal value at baseline and post-intervention (0.16 min [14]).

**Fig. 1:**
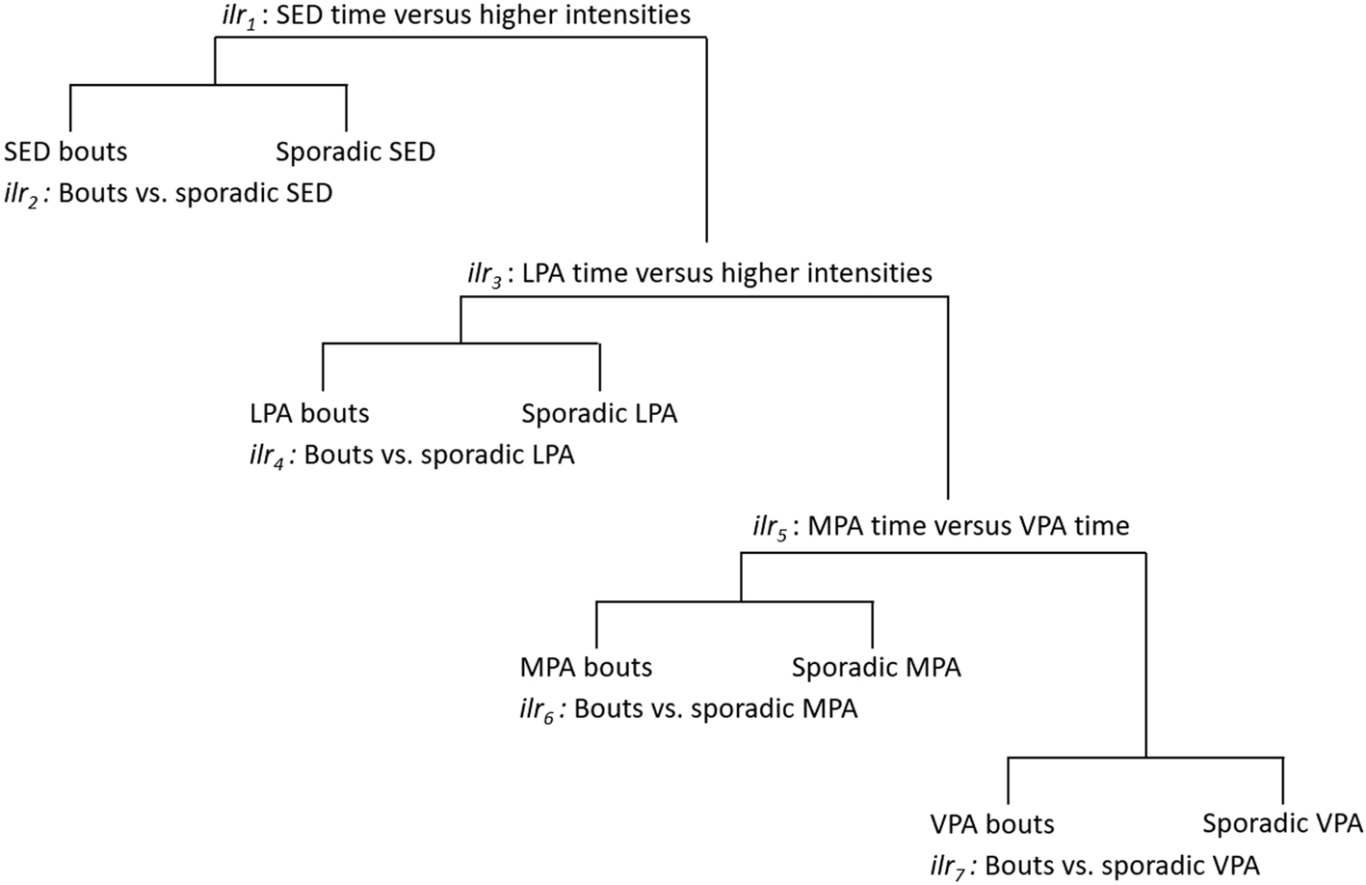
Visual overview of the sequential binary partition

A baseline and post-intervention waking movement behaviour accumulation composition of eight components, including sporadic and bouts of sedentary time, LPA, MPA and VPA, was created using compositional data analysis (*acomp*) [22]. The sequential binary partition with 7 (N components in the waking compositions – 1) *ilr* coordinates was set up so that it enabled comparisons of a) total time spent in one intensity versus others (e.g., total time in sporadic *and* bouts of sedentary time versus higher intensities) and, b) time spent in bouts (e.g., ≥5-min sedentary bouts) versus sporadically accumulated time of a specific intensity. A visual overview of the sequential binary partition is displayed in Fig. 1.

As the changes between baseline and post-intervention are paired data, a “change composition” was calculated with Aitchison’s perturbation method [27–29]. Bar plots of geometric means of the baseline, post-intervention and “change compositions” were used to illustrate the accumulation patterns and changes in these patterns for each intervention group [30]. Linear mixed models were then fitted to assess the associations between intervention groups (as a categorical exposure variable) with the post-intervention composition (multivariate outcome). The initial partially adjusted model accounted for the random effect of individual within each school using default unstructured variance-covariance structure [31, 32] and adjusted for baseline compositions. The fully adjusted model additionally included baseline age (continuous), sex (categorical) and school SES (categorical) as fixed effects. The significance of the explanatory variables was examined with the car::Anova() function [33], which uses Wald Chi squared to calculate Type II tests according to the principle of marginality, testing each covariate after all others [32, 34]. A *p*-value of ≤0.05 was set as the level of statistical significance.

## Results

### Participant characteristics

A flow diagram including the number of included participants from baseline to post-intervention is presented in Fig. S1 in Additional file 1. Further details on reasons for drop-out between baseline and post-intervention are reported in the main outcomes paper (Salmon J, Arundell L, Cerin E, Ridgers ND, Hesketh KD, Daly RM, et al: The Transform-Us! cluster RCT: 18- and 30-month effects on children’s physical activity, sedentary time and cardiometabolic risk markers, unpublished). In total, 267 participants were included in the analytical sample that consisted of those that had valid accelerometry (both timepoints) and covariates data. Participant characteristics of the analytical sample and excluded participants are reported in Table S1 in Additional file 2. No significant and meaningful differences between characteristics of the analytical sample and excluded sample were observed. Although a weekend day was not specified to fulfill valid wear time criteria, 93 and 78% of participants had at least one weekend day of recording at baseline and post-intervention, respectively (Table S1 in Additional file 2). The combined requirements for inclusion in the current analysis (i.e., meet wear time criteria at baseline and post-intervention and have all covariates data) resulted in 61 (23%), 82 (31%), 64 (24%), and 60 (23%) control, PA-I, SB-I and PA + SB-I participants, respectively, to be included. Table S1 in Additional file 2 shows the participants excluded due to < 3 valid days of accelerometer data (*n* = 180 at baseline and *n* = 267 at post-intervention) and due to incomplete covariate data (*n* = 203 at baseline and *n* = 203 at follow-up). Reasonably similar percentages from each group were included. Baseline characteristics (mean ± SD) for the four groups in the analytical sample are presented in Table 1.

**Table 1:** Baseline characteristics for participants in the analytical sample per intervention group (Control, PA-I, SB-I and PA + SB-I)

	Control	N	PA-I	N	SB-I	N	PA + SB-I	N
**Demographics (mean [SD])**								
N (%)	61 (22.8%)	61	82 (30.7%)	82	64 (24.0%)	64	60 (22.5%)	60
Age, years	8.6 (0.3)	61	8.7 (0.4)	82	8.8 (0.4)	64	8.8 (0.3)	60
Sex (n [%])		61		82		64		60
Girls	34 (56%)		45 (55%)		42 (66%)		28 (47%)	
Boys	27 (44%)		37 (45%)		22 (34%)		32 (53%)	
Socio-economic status (n [%])		61		82		64		60
High	0 (0%)		0 (0%)		0 (0%)		9 (15%)	
Mid	38 (62%)		50 (61%)		39 (61%)		36 (60%)	
Low	23 (38%)		32 (39%)		25 (39%)		15 (25%)	
**Adiposity markers (mean [SD])**								
Body mass index, kg/m^2^	17.5 (2.8)	61	17.2 (3.0)	79	17.5 (2.5)	64	17.5 (2.5)	60
Waist circumference, cm	58.8 (6.8)	61	59.3 (7.2)	79	59.9 (6.0)	64	60.5 (5.7)	60
**Accelerometry (mean [SD])**								
Valid weekend days (n [%])^a^		61		82		64		60
No weekend day	3 (4.9%)		7 (8.5%)		3 (4.7%)		7 (11.7%)	
≥ 1 weekend day	58 (95.1%)		75 (91.5%)		61 (95.3%)		53 (88.3%)	
Total SED, min	399.3 (56.0)	61	397.6 (60.2)	82	395.3 (58.5)	64	404.4 (67.6)	60
Total LPA, min	228.6 (34.2)	61	227.1 (31.3)	82	230.2 (37.6)	64	227.2 (36.0)	60
Total MPA, min	57.0 (11.7)	61	53.3 (11.9)	82	53.6 (11.8)	64	58.0 (15.3)	60
Total VPA, min	27.3 (12.0)	61	24.3 (10.3)	82	25.1 (13.0)	64	30.1 (15.7)	60
Time in SED bouts, min^b^	140.0 (40.4)	61	138.9 (47.4)	82	136.9 (49.2)	64	148.8 (54.0)	60
Time in LPA bouts, min^b^	96.3 (23.1)	61	96.0 (20.0)	82	98.3 (25.9)	64	95.0 (23.7)	60
Time in MPA bouts, min^b^	9.8 (4.1)	61	8.5 (3.7)	82	8.2 (3.4)	64	8.8 (4.6)	60
Time in VPA bouts, min^b, c^	7.2 (6.4)	61	6.0 (4.0)	82	6.8 (6.4)	64	8.9 (9.1)	60
MVPA (n [%])^c^		61		82		64		60
< 60 min/day	6 (9.8%)		14 (17.1%)		14 (21.9%)		9 (15.0%)	
≥ 60 min/day	55 (90.2%)		68 (82.9%)		50 (78.1%)		51 (85.0%)	

The analytical sample (total *n* = 267) comprised of participants with complete valid accelerometry and covariates data

Abbreviations: *PA-I* Physical activity intervention group, *SB-I* Sedentary behaviour intervention group, *PA + SB-I* Combined physical activity and sedentary behaviour intervention group, *SD* Standard deviation, *SED* Sedentary time, *LPA* Light-intensity physical activity, *MPA* moderate-intensity physical activity, *VPA* vigorous-intensity physical activity, *MVPA* moderate- to vigorous-intensity physical activity

^a^Valid days were set at a minimum of 8 h on weekdays and 7 h on weekend days

^b^Time in each intensity was divided into sporadic time and time in bouts, using < 5 min and ≥ 5 min for sedentary time, and < 1 min and ≥ 1 min for physical activity (including LPA, MPA and VPA), respectively

^c^Values presented before zero replacement

^d^Based on the average MVPA across included valid days

On average, participants in the analytical sample were 8.7 (SD = 0.4) years old and a higher proportion (56%) were girls than boys. Participants’ mean BMI and waist circumference were 17.4 kg/m^2^ (SD = 2.7 kg/m^2^) and 59.6 cm (SD = 6.5 cm), respectively. The majority of participants (84%) achieved an average of ≥60 minutes of MVPA across included valid days [1].

### The transform-us! Intervention effect movement behaviour accumulation compositions

**Fig. 2:**
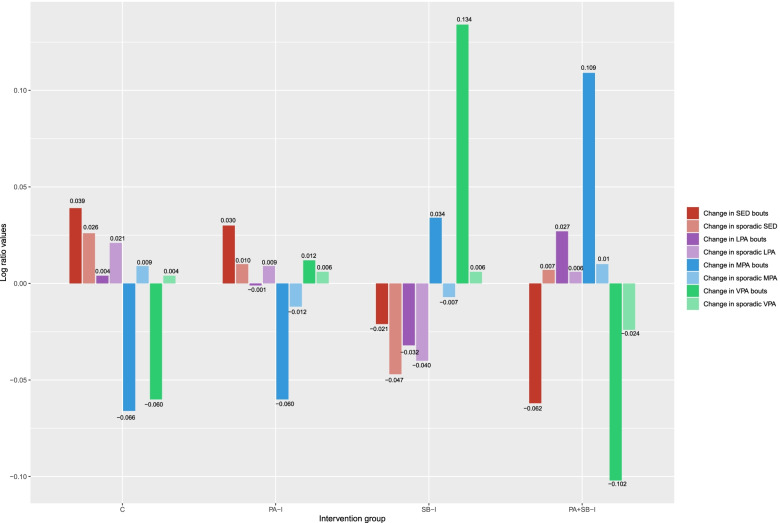
Change between baseline and post−intervention (18 months) in movement behaviour accumulation compositions per intervention group

Figure 2 shows the relative distribution of changes in time spent in sporadic sedentary, LPA, MPA and VPA time and time in bouts of sedentary time, LPA, MPA and VPA, presented as the log-ratios between each group’s compositional mean (also reported in Table S2 in Additional file 3) compared to the overall compositional mean of the complete included sample. Positive and negative bars reflect relative mean values of a part above and below the overall mean “change composition”, respectively. To aid interpretation, the results below are described in terms of the direction of change (i.e., proportional increase or decrease in bouts) and their hypothesized impact on health (i.e., detrimental [assumed for increased time in sedentary bouts and reduced time in MPA/VPA components] or beneficial [assumed for reduced time in sedentary bouts and increased time in MPA/VPA components]). The LPA results are only described with regards to the direction of change as consistent research to justify the direction of contextual interpretation is currently lacking [35].

Figure 2 provides additional insights beyond the absolute changes in time in bouts of and sporadic sedentary, LPA, MPA, and VPA time from baseline to post-intervention that are detailed in Table S2 in Additional file 3. For example, the absolute values in Table S2 in Additional file 3 show that all groups decreased their time in both sporadic and bouts of VPA. However, Fig. 2 shows that the SB-I group had the smallest proportional decrease in VPA bouts over time (i.e., beneficial change) compared to the overall compositional sample mean of change. The SB-I group was also characterized by the largest proportional decrease of time in sporadic LPA, and smallest proportional increase of time in LPA bouts, compared to the overall compositional mean. The combined intervention group (PA + SB-I) was characterized by the largest proportional increase in time in MPA bouts (i.e., beneficial change). The usual practice group was characterized by the largest proportional increase in both sporadic sedentary time and time in sedentary bouts; detrimental change).

Whilst the graphical representation of “change compositions” appeared to differ across groups (Fig. 2), the analysis of variance of both the partially adjusted and fully adjusted linear mixed model parameters indicated that overall, the intervention group post-intervention movement behaviour accumulation compositions, and most of the individual *ilr* coordinates within these, were not statistically significantly different from the control group (Tables 2 and 3). Plots for the separate baseline and post-intervention movement behaviour accumulation compositions are shown in Figs. S2-S3 (Additional file 4). Baseline, post-intervention, and “change compositions” for the total volumes (i.e., time not divided in sporadic time and time in bouts bouts) are presented in Additional file 4 in Fig. S4-S6.

**Table 2:** Linear mixed models for the intervention effects on post-intervention movement behaviour accumulation composition (*n* = 267)

	Chi square	Df	***p***-value
**Partially adjusted model**			
Intervention group	4.020	21	> 0.999
**Fully adjusted model**			
Intervention group	5.889	21	> 0.999
Age	23.555	7	0.001*
Sex	65.392	7	< 0.001*
Socio-economic status	3.762	14	0.997

Linear mixed models with analysis of variance Type II Wald Chi square assessed associations between intervention groups and post-intervention movement behaviour accumulation compositions (multivariate outcome)

Partially adjusted models accounted for the random effect of individual within each school and adjusted for baseline movement behaviour accumulation compositions. Adjusted models additionally included baseline age (continuous), sex (categorical) and SES (categorical) as fixed effects

Abbreviation: *Df* Degrees of Freedom

*Denote statistical significance at the *p* < 0.05 level

**Table 3:** Linear mixed models for the intervention effects on individual *ilr* coordinates within the post-intervention movement behaviour accumulation compositions (*n* = 267)

	Partially adjusted model		Fully adjusted model	
	β (SE)	***p***-value	β (SE)	***p***-value
***ilr***_***1***_: **Total SED time (including sporadic SED and time in SED bouts) versus time in higher intensities**	0.88 (0.28)	< 0.01*	−1.39 (0.77)	0.07
***ilr***_***2:***_ **Time in SED bouts versus sporadic SED time**	−0.44 (0.26)	0.09	−1.18 (0.72)	0.10
***ilr***_***3:***_ **Total LPA time (including sporadic LPA and time in LPA bouts) versus time in higher intensities**	0.98 (0.28)	< 0.01*	− 0.83 (0.79)	0.30
***ilr***_***4:***_ **Time in LPA bouts versus sporadic LPA time**	− 0.09 (0.26)	0.74	− 0.44 (0.72)	0.54
***ilr***_***5:***_ **Total MPA time (including sporadic MPA and time in MPA bouts) versus total VPA time (including sporadic VPA and time in VPA bouts)**	0.46 (0.29)	0.11	−1.12 (0.82)	0.18
***ilr***_***6:***_ **Sporadic MPA versus time in MPA bouts**	−0.42 (0.27)	0.12	−1.08 (0.75)	0.15
***ilr***_***7:***_ **Sporadic VPA versus time in VPA bouts**	−0.51 (0.28)	0.07	−0.06 (0.80)	0.94

A visual representation of the *ilr* coordinates is provided in Fig. 1

Time in each intensity was divided into sporadic time and time in bouts, using < 5 min and ≥ 5 min for sedentary time, and < 1 min and ≥ 1 min for physical activity (including LPA, MPA and VPA), respectively

Linear mixed models with analysis of variance Type II Wald Chi square assessed associations between intervention groups and post-intervention movement behaviour accumulation compositions (multivariate outcome)

Partially adjusted models accounted for the random effect of individual within each school and adjusted for baseline movement behaviour accumulation compositions. Adjusted models additionally included baseline age (continuous), sex (categorical) and SES (categorical) as fixed effects

Abbreviations: *SE* Standard Error

^*^Denote statistical significance at the *p* < 0.05 level

## Discussion

This study aimed to investigate effects of the *Transform-Us!* school- and home-based intervention program on primary school children’s movement behaviour accumulation patterns, including sporadic and bouts of sedentary time and physical activity. Intervention effects on the distribution of time in the waking movement behaviour accumulation composition were small and not statistically significant. However, visual inspection revealed that the groups with no sedentary behaviour intervention component (PA-I and control) demonstrated an increase in sedentary bouts, while the other two groups (SB-I and PA + SB-I) exhibited a reduction of time in sedentary bouts, compared to the overall sample compositional mean. In addition, the SB-I group was the only group with an increase in VPA over time.

Previous intervention studies have had limited success in changing movement behaviours in children, with systematic reviews reporting only a small increase in MVPA (approximately 5 min/day) from school-based physical activity interventions and no convincing evidence for the effectiveness of sedentary behaviour interventions [7, 36]. Whilst the present analysis specifically focused on the accumulation of movement behaviours, rather than the total volume, it did show that the SB-I and the SB + PA-I intervention groups had the most favourable changes in total sedentary time and MVPA, compared to the groups without a sedentary intervention component. Whilst this is consistent with the *Transform-Us!* main outcomes paper that showed improved MVPA levels in children who received the SB intervention compared to those who did not (Salmon J, Arundell L, Cerin E, Ridgers ND, Hesketh KD, Daly RM, et al: The Transform-Us! cluster RCT: 18- and 30-month effects on children’s physical activity, sedentary time and cardiometabolic risk markers, unpublished), it is somewhat unexpected that the sedentary behaviour intervention component, as opposed to the physical activity intervention component, may have led to increased activity levels.

While differences in movement behaviour accumulation patterns between groups were small and not significant, the two groups with the largest increases in physical activity were the SB-I and PA + SB-I groups. Whereas the SB-I group demonstrated the most favourable changes in VPA bouts over time, the PA + SB-I group demonstrated this in MPA bouts. It is possible that children in the SB-I group were naturally stimulated to engage in more VPA due to the short duration of the activity breaks, while the PA + SB-I group may have been encouraged to do more MPA during the longer physical activity intervention components. Given previous evidence suggests that stronger associations with cardio-metabolic health benefits in children may be obtained by more vigorous-intensity activities through such time-economic high-intensity bouts [37], compared to continuous behaviours, the focus on such short intervention components rather than longer ones may be promising to increase physical activity intensity and thereby improve health. Future studies will need to investigate which intervention components are most effective in increasing vigorous-intensity physical activity levels.

Another potential explanation for the observed differences in changes over time may be that the physical activity strategies of the *Transform-Us!* intervention aimed to increase a behaviour that participants were already doing, though at varying durations, whereas the sedentary behaviour strategies aimed at reducing sitting time and replacing it with activity (e.g., stand or move). As children will rarely stand still [38], this may have contributed to more physical activity in the sedentary behaviour intervention groups, despite this not being targeted specifically. It is possible that the “break up your sitting” message may be more impactful than the “move more” message to target movement behaviours in this age group. Results from a systematic review of intervention strategies that exclusively target interruptions to sitting time showed that it is currently unclear whether intervention components are effective in changing movement behaviours [7], hence, future research should investigate if this type of messaging is indeed more effective in changing movement behaviours and consequently health in youth. This information is also warranted for informing updates in current movement behaviour recommendations, which mostly focus on overall time in movement behaviours, to incorporate specific accumulation patterns (e.g., bout duration recommendations).

The present work has several limitations that need to be acknowledged. Firstly, the small sample size may have impacted the ability to detect significant intervention effects over time. It is inappropriate to conduct a retrospective sample size calculation [39], as the *Transform-Us!* trial was not originally powered for the secondary analysis conducted. In addition, power calculations for compositional data analysis have been proven difficult due to the novelty of dealing with multivariate nature of the compositional variables in behavioural research [9]. Secondly, > 80% of the sample engaged in ≥60 minutes of MVPA, on average, per day, which is more than generally observed (15–41%) in Australian youth using self-reported measures [40]. The findings may therefore not be generalizable to the wider population, and these should be verified in less active and perhaps more representative samples. Thirdly, it is widely accepted that there is some measurement error associated with the use of ActiGraph accelerometers. This is particularly relevant for the assessment of sedentary time, as the ActiGraph estimates sedentary time is based on a lack of movement, and therefore cannot distinguish between sitting (i.e., sedentary time) and standing still (i.e., LPA) [41]. Finally, the current study did not collect 24-hour data (i.e., only waking behaviours were assessed), which limits full understanding of child movement behaviours [1]. Future studies should include larger sample sizes to confirm or counter the findings in the present work. These studies should preferably use 24-hour designs, using posture-based devices including accelerometers, and focus on more representative samples.

## Conclusion

This study was the first to use compositional data analysis on children’s objective data to investigate intervention effects on movement behaviour accumulation patterns. Intervention effects on the distribution of time in the waking movement behaviour accumulation composition were small and not statistically significant. However, visual inspection of the change in compositions over time revealed that only groups with a sedentary behaviour intervention component (SB-I and PA + SB-I) reduced the time in sedentary bouts, compared to the overall sample compositional mean. In addition, the SB-I group was the only group with an increase in vigorous-intensity physical activity and the combined intervention group (PA + SB-I) was characterized by the largest proportional increase in MPA bouts. Overall, this study showed some early evidence to suggest that the “break up your sitting” message may be more impactful than the “move more” message in this age group. Future research, including larger sample sizes and including posture-based devices, should investigate if this type of messaging is indeed more effective in changing movement behaviours and ultimately child health.

## Supplementary Information

**Additional file 1: Figure S1.** Participant flow diagram.

**Additional file 2: Table S1.** Participant characteristics of the analytical sample (*n* = 267) and excluded participants.

**Additional file 3: Table S2.** Absolute and proportional time in total, sporadic and bouts of SED, LPA, MPA, and VPA at baseline and post-intervention per intervention group.

**Additional file 4: Figure S2.** Baseline movement behaviour accumulation compositions per intervention group. **Figure S3.** Post-intervention (18 months) movement behaviour accumulation compositions per intervention group. **Figure S4.** Baseline total volume compositions per intervention group. **Figure S5.** Post-intervention (18 months) total volume compositions per intervention group. **Figure S6.** Change between baseline and post-intervention (18 months) in total volume compositions per intervention group.

## Data Availability

Data will not be shared as this was secondary data analysis. No datasets were generated for the current study. The authors have no permission to share the original datasets. For queries regarding the Transform-Us! dataset please contact the principal investigator via email: jo.salmon@deakin.edu.au.
