# Supplementary material for: Intervention effects on children’s movement behaviour accumulation as a result of the Transform-Us! school- and home-based cluster randomised controlled trial
Source: Int J Behav Nutr Phys Act. 2022 Jul 7;19:76. doi: 10.1186/s12966-022-01314-z (PMC9261108; doi:10.1186/s12966-022-01314-z)
Supplement: Supplementary file 1 — Additional file 1: Figure S1. Participant flow diagram. [file 12966_2022_1314_MOESM1_ESM.docx]

**ADDITIONAL FILE 1**

**Figure 1. Participant flow diagram**

Excluded

Did not meet Year 3 inclusion criteria (n=4)

Declined to participate (n=49)

No response (n=960)

Schools invited to participate

(n=127)

(Low SES=41; Mid SES=45; High-SES=41)

Schools recruited

(n=20)

(Low SES=8; Mid SES=11; High SES=1)

Children eligible to participate

(n=1606)

Randomised

(n=20 schools)

ENROLMENT

ALLOCATION

ANALYSIS

FOLLOW-UP

**Current practice control**

5 schools

Allocated to intervention

(n=149 children)

Received allocated intervention

(n=148)

Did not receive allocated

intervention

Left school (n=1)

**PA-I**

5 schools

Allocated to intervention

(n=161 children)

Received allocated intervention

(n=160)

Did not receive allocated

intervention

Left school (n= 1)

**SB-I**

5 schools

Allocated to intervention

(n=124 children)

Received allocated intervention

(n=124)

**PA+SB-I**

5 schools

Allocated to intervention

(n=159 children)

Received allocated intervention

(n=159)

**Current practice control**

5 schools

Lost to follow-up

18-month assessments:

Left school (n=9)

Dropped out (n=2)

30-month assessments:

Left school (n=9)

Dropped out (n=6)

**PA-I**

5 schools

Lost to follow-up

18-month assessments:

Left school (n=11)

Dropped out (n=4)

30-month assessments:

Left school (n=9)

Dropped out (n=9)

**SB-I**

5 schools

Lost to follow-up

18-month assessments:

Left school (n=7)

Dropped out (n=2)

30-month assessments:

Left school (n=10)

Dropped out (n=8)

**PA+SB-I**

5 schools

Lost to follow-up

18-month assessments:

Left school (n=18)

Dropped out (n=5)

30-month assessments:

Left school (n=27)

Dropped out (n=5)

**Current practice control**

Included in analytical sample*

(n=61)

**PA-I**

Included in analytical sample*

(n=82)

**SB-I**

Included in analytical sample*

(n=64)

**PA+SB-I**

Included in analytical sample*

(n=60)

Abbreviations: SES: Socio-economic status; PA-I: Physical activity intervention group; SB-I: Sedentary behaviour intervention group; PA+SB-I: Combined physical activity and sedentary behaviour intervention group.

*The analytical sample (total n=267) comprised of participants with complete valid accelerometry and covariates data.
