# Supplementary material for: Intervention effects on children’s movement behaviour accumulation as a result of the Transform-Us! school- and home-based cluster randomised controlled trial
Source: Int J Behav Nutr Phys Act. 2022 Jul 7;19:76. doi: 10.1186/s12966-022-01314-z (PMC9261108; doi:10.1186/s12966-022-01314-z)
Supplement: Supplementary file 2 — Additional file 2: Table S1. Participant characteristics of the analytical sample (n = 267) and excluded participants. [file 12966_2022_1314_MOESM2_ESM.docx]

**ADDITIONAL FILE 2**

| **Table S1. Participant characteristics of the analytical sample (n=267) and excluded participants** | | | | | | | | | | | | | | | | | |
| --- | --- | --- | --- | --- | --- | --- | --- | --- | --- | --- | --- | --- | --- | --- | --- | --- | --- |
| **Demographics (measured at baseline; mean [SD])** | | | | | | | | | | | | | | | | | |
|  | | **Analytical sample** | | | | **N** | | | **Excluded participants** | | | | **N** | | | $\boldsymbol{p}$**-value** | |
| Intervention group (n [%]) | |  | | | | 267 | | |  | | | | 383 | | |  | |
| Control | | 61 (22.8%) | | | |  | | | 102 (26.6%) | | | |  | | |  | |
| PA-I | | 82 (30.7%) | | | |  | | | 99 (25.8%) | | | |  | | |  | |
| SB-I | | 64 (24.0%) | | | |  | | | 75 (19.6%) | | | |  | | |  | |
| PA+SB-I | | 60 (22.5%) | | | |  | | | 107 (27.9%) | | | |  | | |  | |
| Age, years | | 8.7 (0.4) | | | | 267 | | | 8.7 (0.4) | | | | 309 | | | 0.555 | |
| Sex (n [%]) | |  | | | | 267 | | |  | | | | 370 | | | 0.394 | |
| Girls | | 149 (55.8%) | | | |  | | | 219 (57.2%) | | | |  | | |  | |
| Boys | | 118 (44.2%) | | | |  | | | 151 (39.4%) | | | |  | | |  | |
| Socio-economic status (n [%]) | |  | | | | 267 | | |  | | | | 383 | | | 0.462 | |
| High | | 9 (3.4%) | | | |  | | | 9 (2.3%) | | | |  | | |  | |
| Mid | | 163 (61.0%) | | | |  | | | 222 (58.0%) | | | |  | | |  | |
| Low | | 95 (35.6%) | | | |  | | | 152 (39.7%) | | | |  | | |  | |
| **Adiposity markers (measured at baseline; mean [SD])** | | | | | | | | | | | | | | | | | |
| Body mass index, kg/m^2^ | | 17.4 (2.7) | | | | 264 | | | 18.4 (3.1) | | | | 264 | | | 0.1948 | |
| Waist circumference, cm | | 59.6 (6.5) | | | | 264 | | | 62.2 (7.2) | | | | 265 | | | 0.2934 | |
|  | |  | |  | | |  | | | |  | | | |  | | |
|  | |  | |  | | |  | | | |  | | | |  | | |
| **Accelerometry (measured at baseline and post-intervention, mean [SD])** | | | | | | | | | | | | | | | | | |
|  | **BASELINE** | | | | | | | | | **POST-INTERVENTION (18 MONTHS)** | | | | | | | |
|  | **Analytical sample** | | **N** | | **Excluded participants** | | | **N** | | **Analytical sample** | | **N** | | **Excluded participants** | | | **N** |
| Valid days (n [%])^A^ |  | | 267 | |  | | | 383 | |  | | 267 | |  | | | 383 |
| <3 days | 0 (0.0%) | |  | | 180 (47.0%) | | |  | | 0 (0.0%) | |  | | 267 (69.7%) | | |  |
| ≥3 days | 267 (100.0%) | |  | | 203 (53.0%) | | |  | | 267 (100.0%) | |  | | 203 (30.3%) | | |  |
| Valid weekend days (n [%])^A^ |  | | 267 | |  | | | 383 | |  | | 267 | |  | | | 383 |
| No weekend day | 20 (7.5%) | |  | | 196 (51.2%) | | |  | | 60 (22.5%) | |  | | 283 (73.9%) | | |  |
| ≥1 weekend day | 247 (92.5%) | |  | | 187 (48.8%) | | |  | | 207 (77.5%) | |  | | 100 (26.1%) | | |  |
| Total SED, min | 399.0 (60.4) | | 267 | | 386.8 (64.3) | | | 132 | | 418.9 (66.4) | | 267 | | 423.6 (75.2) | | | 115 |
| Total LPA, min | 228.2 (34.5) | | 267 | | 226.8 (33.6) | | | 132 | | 233.9 (34.1) | | 267 | | 231.1 (38.4) | | | 115 |
| Total MPA, min | 55.3 (12.8) | | 267 | | 55.2 (13.6) | | | 132 | | 47.1 (13.3) | | 267 | | 47.2 (12.5) | | | 115 |
| Total VPA, min | 26.5 (12.8) | | 267 | | 26.8 (12.0) | | | 132 | | 21.8 (12.9) | | 267 | | 21.9 (12.1) | | | 115 |
| Time in SED bouts, min^B^ | 140.9 (47.9) | | 267 | | 133.6 (49.9) | | | 132 | | 151.4 (53.1) | | 267 | | 156.3 (59.5) | | | 115 |
| Time in LPA bouts, min^B^ | 96.4 (23.0) | | 267 | | 95.7 (22.4) | | | 132 | | 105.3 (22.4) | | 267 | | 102.6 (26.2) | | | 115 |
| Time in MPA bouts, min^B^ | 8.8 (4.0) | | 267 | | 8.4 (4.1) | | | 132 | | 8.4 (4.3) | | 267 | | 8.2 (4.4) | | | 115 |
| Time in VPA bouts, min^B, C^ | 7.1 (6.6) | | 267 | | 6.7 (5.0) | | | 132 | | 6.4 (6.3) | | 267 | | 6.4 (6.9) | | | 115 |
| MVPA (n [%])^D^ |  | | 267 | |  | | | 383 | |  | | 267 | |  | | | 383 |
| <60 min/day | 43 (16.1%) | |  | | 20 (5.2%) | | |  | | 114 (42.7%) | |  | | 45 (11.7%) | | |  |
| ≥60 min/day | 224 (83.9%) | |  | | 363 (94.8%) | | |  | | 153 (57.3%) | |  | | 338 (88.3%) | | |  |
| The analytical sample (total n=267) comprised of participants with complete valid accelerometry and covariates data.  Participant characteristics were compared using t-tests (continuous variables) and chi-square tests (categorical variables).  ^A^ Valid days were set at a minimum of 8 hours on weekdays and 7 hours on weekend days.  ^B^ Time in each intensity was divided into sporadic time and time in bouts, using <5 min and ≥5 min for sedentary time, and <1 min and ≥1 min for physical activity (including LPA, MPA and VPA), respectively.  ^C^ Values presented before zero replacement.  ^D^ Based on the average MVPA across included valid days.  Abbreviations: SD: Standard deviation; PA-I: Physical activity intervention group; SB-I: Sedentary behaviour intervention group; PA+SB-I: Combined physical activity and sedentary behaviour intervention group; SED: Sedentary time; LPA: Light-intensity physical activity; MPA: moderate-intensity physical activity; VPA: vigorous-intensity physical activity; MVPA: moderate- to vigorous-intensity physical activity. | | | | | | | | | | | | | | | | | |
