# Supplementary material for: Intervention effects on children’s movement behaviour accumulation as a result of the Transform-Us! school- and home-based cluster randomised controlled trial
Source: Int J Behav Nutr Phys Act. 2022 Jul 7;19:76. doi: 10.1186/s12966-022-01314-z (PMC9261108; doi:10.1186/s12966-022-01314-z)
Supplement: Supplementary file 3 — Additional file 3: Table S2. Absolute and proportional time in total, sporadic and bouts of SED, LPA, MPA, and VPA at baseline and post-intervention per intervention group. [file 12966_2022_1314_MOESM3_ESM.docx]

**ADDITIONAL FILE 3**

| **Table S2.** **Absolute and proportional time in total, sporadic and bouts of SED, LPA, MPA, and VPA at baseline and post-intervention per intervention group** | | | | | | | | | | | | | | | | |
| --- | --- | --- | --- | --- | --- | --- | --- | --- | --- | --- | --- | --- | --- | --- | --- | --- |
|  | **Control (N=61)** | | | | | **PA-I (N=82)** | | | | **SB-I (N=64)** | | | **PA+SB-I (N=60)** | | | |
|  | **Baseline**^A^ | | **Post-IV^B^** | **Change** | **Baseline**^A^ | | **Post-IV^B^** | **Change** | **Baseline**^A^ | | **Post-IV^B^** | **Change** | | **Baseline**^A^ | **Post-IV^B^** | **Change** |
|  | Mean, min *% wear* | | Mean, min *% wear* | Geometric mean | Mean, min *% wear* | | Mean, min *% wear* | Geometric mean | Mean, min *% wear* | | Mean, min *% wear* | Geometric mean | | Mean, min *% wear* | Mean, min *% wear* | Geometric mean |
| **Accumulation patterns** | | **Bout pattern time-use compositions** | | | | | | | | | | | | | | |
| SED bouts | 136.64 | | 149.98 | 0.15 | 135.15 | | 148.40 | 0.15 | 132.56 | | 144.60 | 0.14 | | 141.83 | 144.51 | 0.14 |
|  | *19.27%* | | *20.78% ↑* |  | *19.06%* | | *20.56% ↑* |  | *18.70%* | | *20.04% ↑* |  | | *20.01%* | *20.03% ↑* |  |
| Sporadic SED | 262.00 | | 273.01 | 0.14 | 265.93 | | 275.41 | 0.14 | 266.12 | | 271.96 | 0.13 | | 257.70 | 270.63 | 0.14 |
|  | *36.96%* | | *37.83% ↑* |  | *37.51%* | | *38.16% ↑* |  | *37.54%* | | *37.69% ↑* |  | | *36.35%* | *37.50% ↑* |  |
| LPA bouts | 95.25 | | 103.16 | 0.15 | 97.22 | | 105.81 | 0.15 | 98.48 | | 108.57 | 0.14 | | 93.77 | 106.73 | 0.15 |
|  | *13.44%* | | *14.29% ↑* |  | *13.71%* | | *14.66% ↑* |  | *13.89%* | | *15.05% ↑* |  | | *13.23%* | *14.79% ↑* |  |
| Sporadic LPA | 133.80 | | 130.75 | 0.13 | 134.85 | | 131.43 | 0.13 | 135.82 | | 131.70 | 0.13 | | 133.28 | 131.71 | 0.13 |
|  | *18.87%* | | *18.12% ↓* |  | *19.02%* | | *18.21% ↓* |  | *19.16%* | | *18.25% ↓* |  | | *18.80%* | *18.25% ↓* |  |
| MPA bouts | 9.19 | | 7.87 | 0.12 | 8.01 | | 6.97 | 0.12 | 7.80 | | 7.79 | 0.13 | | 7.82 | 8.20 | 0.14 |
|  | *1.30%* | | *1.09% ↓* |  | *1.13%* | | *0.97% ↓* |  | *1.10%* | | *1.08% ↓* |  | | *1.10%* | *1.14% ↑* |  |
| Sporadic MPA | 47.16 | | 38.39 | 0.11 | 45.26 | | 36.43 | 0.11 | 46.01 | | 38.90 | 0.11 | | 48.51 | 40.61 | 0.11 |
|  | *6.65%* | | *5.32% ↓* |  | *6.38%* | | *5.05% ↓* |  | *6.49%* | | *5.39% ↓* |  | | *6.84%* | *5.63% ↓* |  |
| VPA bouts | 5.53 | | 3.98 | 0.10 | 4.86 | | 3.80 | 0.11 | 4.51 | | 4.16 | 0.12 | | 5.97 | 4.24 | 0.09 |
|  | *0.78%* | | *0.55% ↓* |  | *0.69%* | | *0.53% ↓* |  | *0.64%* | | *0.58% ↓* |  | | *0.84%* | *0.59% ↓* |  |
| Sporadic VPA | 19.37 | | 14.52 | 0.10 | 17.66 | | 13.39 | 0.10 | 17.64 | | 13.96 | 0.10 | | 20.07 | 15.01 | 0.10 |
|  | *2.73%* | | *2.01% ↓* |  | *2.49%* | | *1.86% ↓* |  | *2.49%* | | *1.93% ↓* |  | | *2.83%* | *2.08% ↓* |  |
| **Total volumes** | | **Total volume time-use compositions** | | | | | | | | | | | | | | |
| Total SED | 398.68 | | 423.67 | 0.37 | 402.22 | | 424.67 | 0.37 | 399.50 | | 417.06 | 0.36 | | 400.07 | 414.95 | 0.35 |
|  | *56.24%* | | *58.71% ↑* |  | *56.74%* | | *58.85% ↑* |  | *56.35%* | | *57.79% ↑* |  | | *56.43%* | *57.50% ↑* |  |
| Total LPA | 227.79 | | 231.57 | 0.35 | 230.00 | | 234.84 | 0.35 | 232.06 | | 237.81 | 0.35 | | 225.04 | 236.22 | 0.36 |
|  | *32.13%* | | *32.09% ↓* |  | *32.44%* | | *32.54% ↑* |  | *32.73%* | | *32.95% ↑* |  | | *31.74%* | *32.73% ↑* |  |
| Total MVPA | 82.47 | | 66.41 | 0.28 | 76.72 | | 62.14 | 0.28 | 77.38 | | 66.78 | 0.29 | | 83.83 | 70.48 | 0.29 |
|  | *11.63%* | | *9.20% ↓* |  | *10.82%* | | *8.61% ↓* |  | *10.91%* | | *9.25% ↓* |  | | *11.82%* | *9.77% ↓* |  |
| The analytical sample (total n=267) comprised of participants with complete valid accelerometry and covariates data.  Time in each intensity was divided into sporadic time and time in bouts, using <5 min and ≥5 min for sedentary time, and <1 min and ≥1 min for physical activity (including LPA, MPA and VPA), respectively.  The group geometric means are coherent with the inter-dependent nature of compositional data and represent the proportional change in components between baseline and post-intervention. These are compared with the overall sample geometric mean in Figure 2 and Additional file 4: Figures S2-S6.  ^A^ Calculated from compositional proportional time using the baseline total wear of 708.9 minutes.  ^B^ Calculated from compositional proportional time using the post-intervention total wear of 721.6 minutes.  ↑ Increase in proportional time in pattern from baseline to post-intervention (in % of the weartime).  ↓ Decrease in proportional time in pattern from baseline to post-intervention (in % of the weartime).  Abbreviations: SED: Sedentary time; LPA: Light-intensity physical activity; MPA: moderate-intensity physical activity; VPA: vigorous-intensity physical activity; PA-I: Physical activity intervention group; SB-I: Sedentary behaviour intervention group; PA+SB-I: Combined physical activity and sedentary behaviour intervention group; Post-IV: Post-intervention; MVPA: moderate- to vigorous-intensity physical activity. | | | | | | | | | | | | | | | | |
