# Supplementary material for: Intervention effects on children’s movement behaviour accumulation as a result of the Transform-Us! school- and home-based cluster randomised controlled trial
Source: Int J Behav Nutr Phys Act. 2022 Jul 7;19:76. doi: 10.1186/s12966-022-01314-z (PMC9261108; doi:10.1186/s12966-022-01314-z)

ADDITIONAL FILE 4

Figure S2. Baseline movement behaviour accumulation compositions per intervention group

Figure S3. Post-intervention (18 months) movement behaviour accumulation compositions per intervention group

Figure S4. Baseline total volume compositions per intervention group

Figure S5. Post-intervention (18 months) total volume compositions per intervention group

Figure S6. Change between baseline and post-intervention (18 months) in total volume compositions per intervention group

Figure S2. Baseline movement behaviour accumulation compositions per intervention group

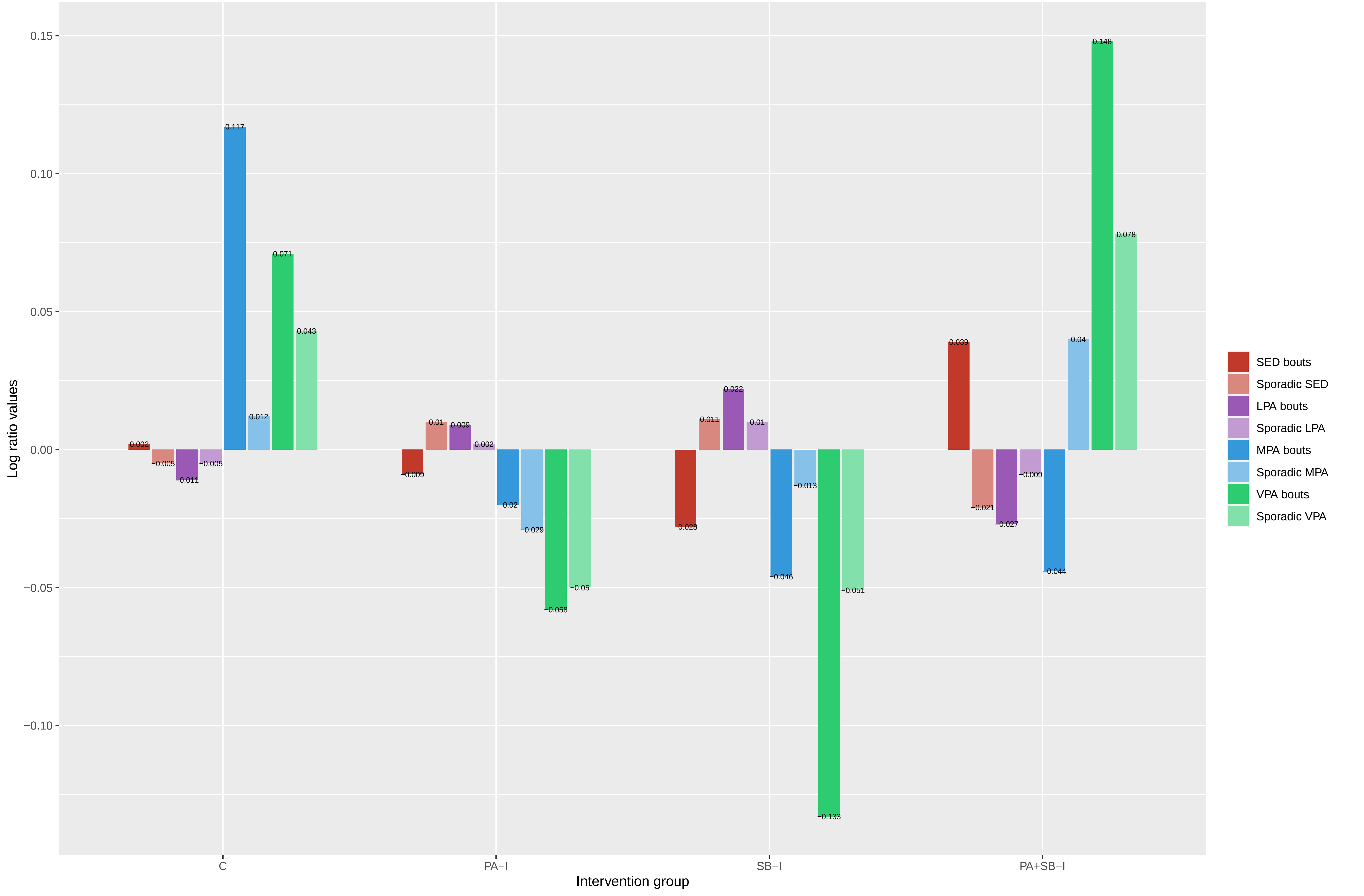

Figure S3. Follow−up (18 months) movement behaviour accumulation compositions per intervention group

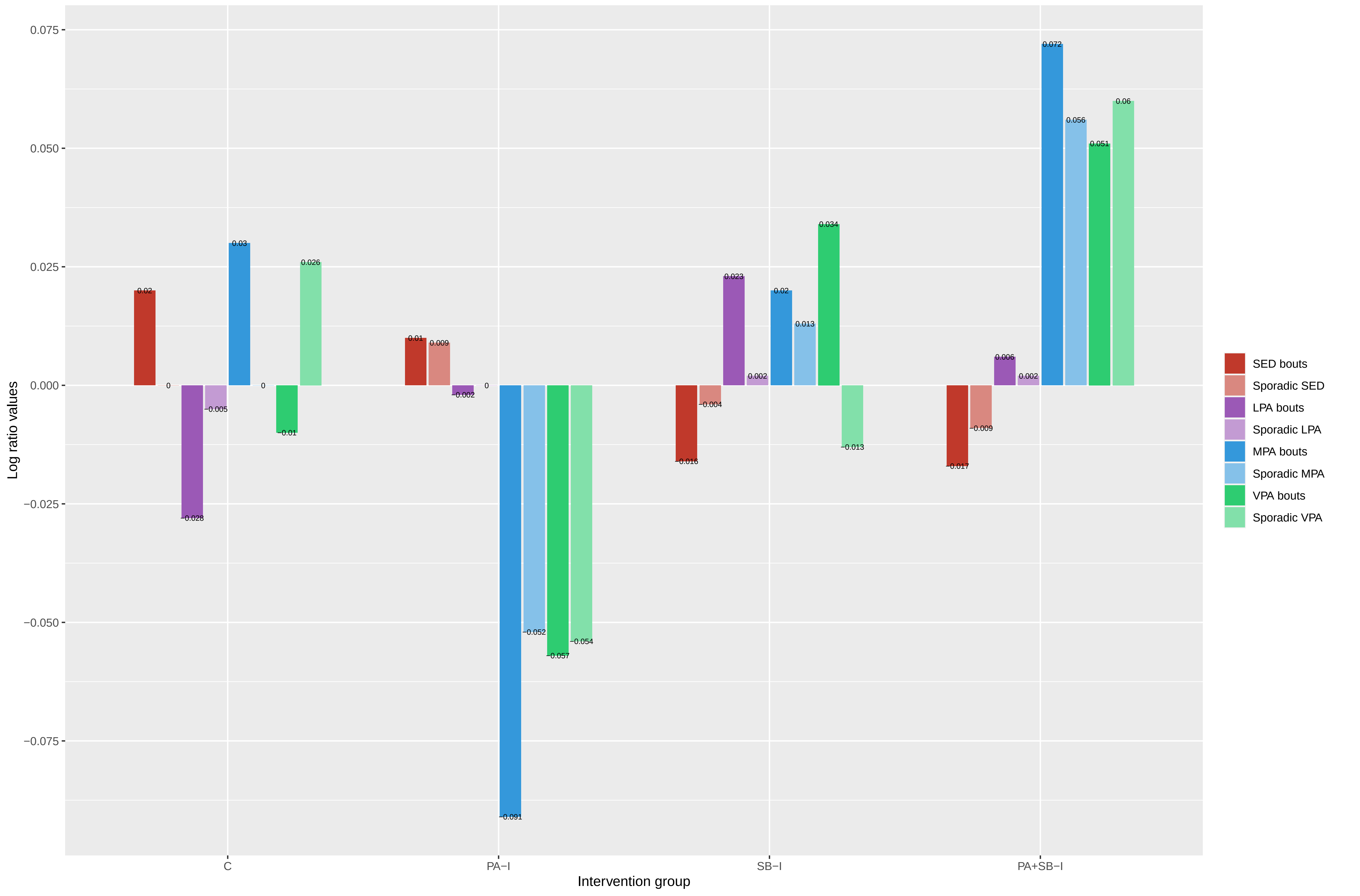

Figure S4. Baseline total volume compositions per intervention group

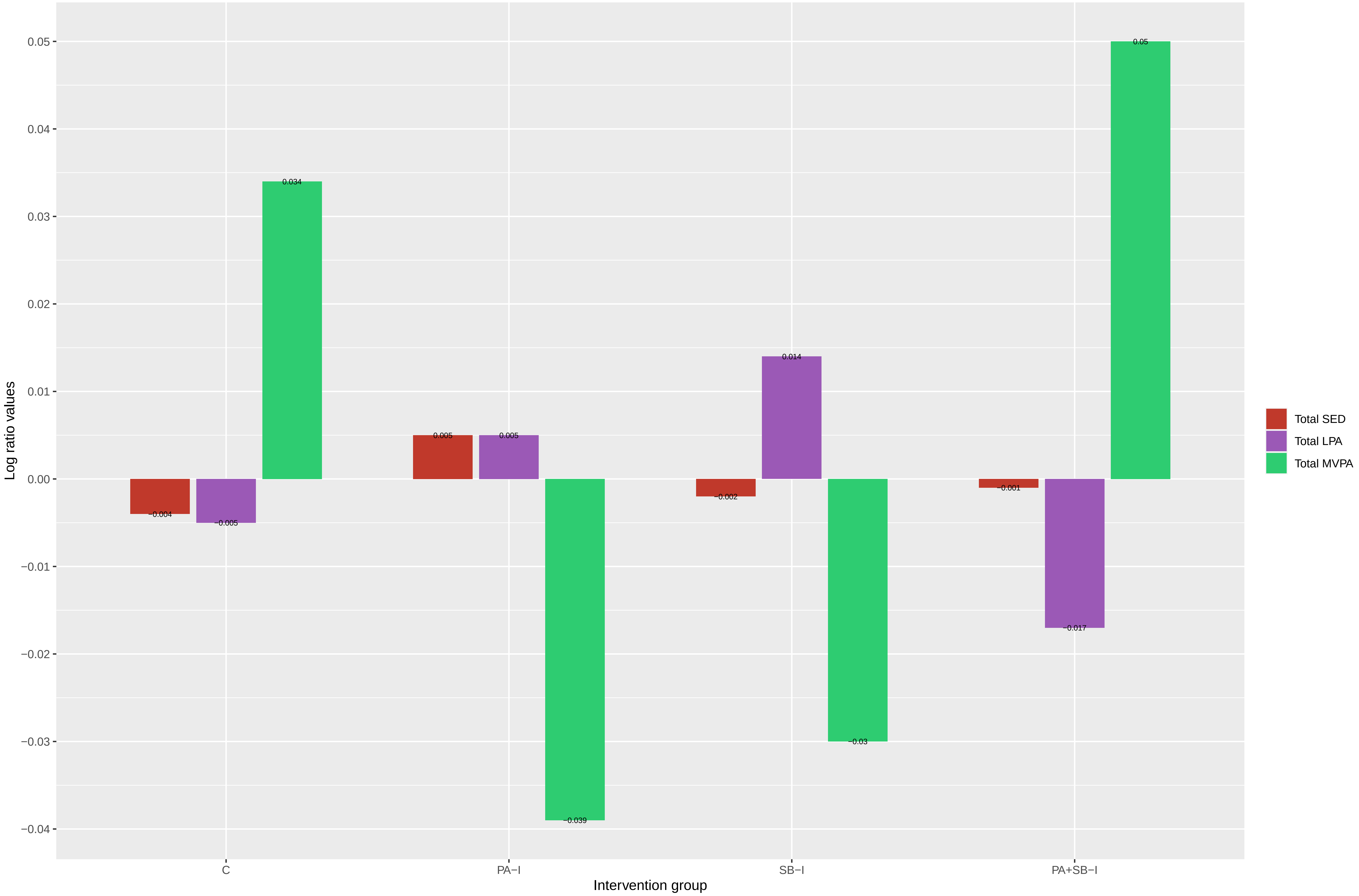

Figure S5. Post−intervention (18 months) total volume compositions per intervention group

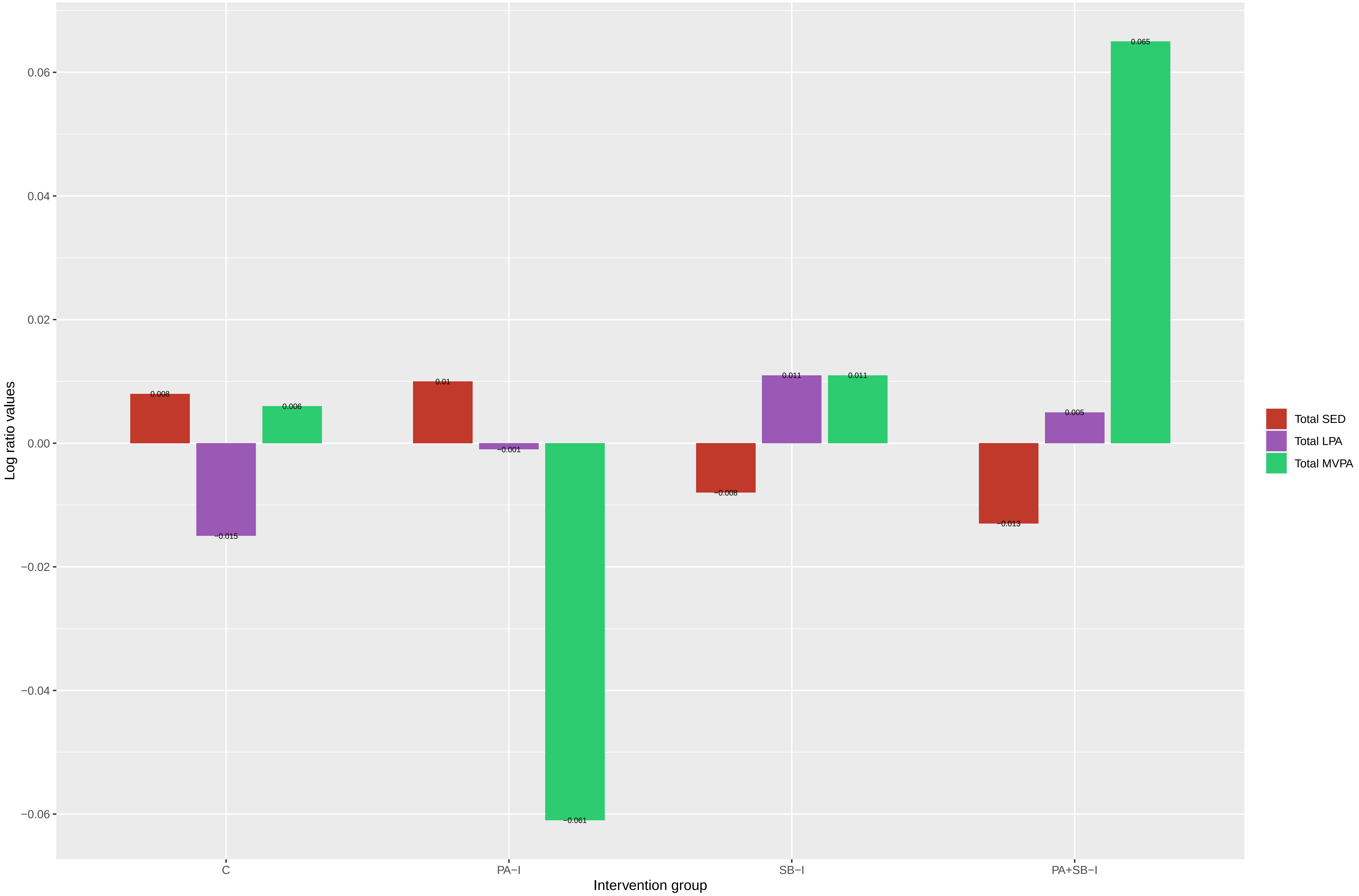

Figure S6. Change between baseline and post−intervention (18 months) in total volume compositions per intervention group

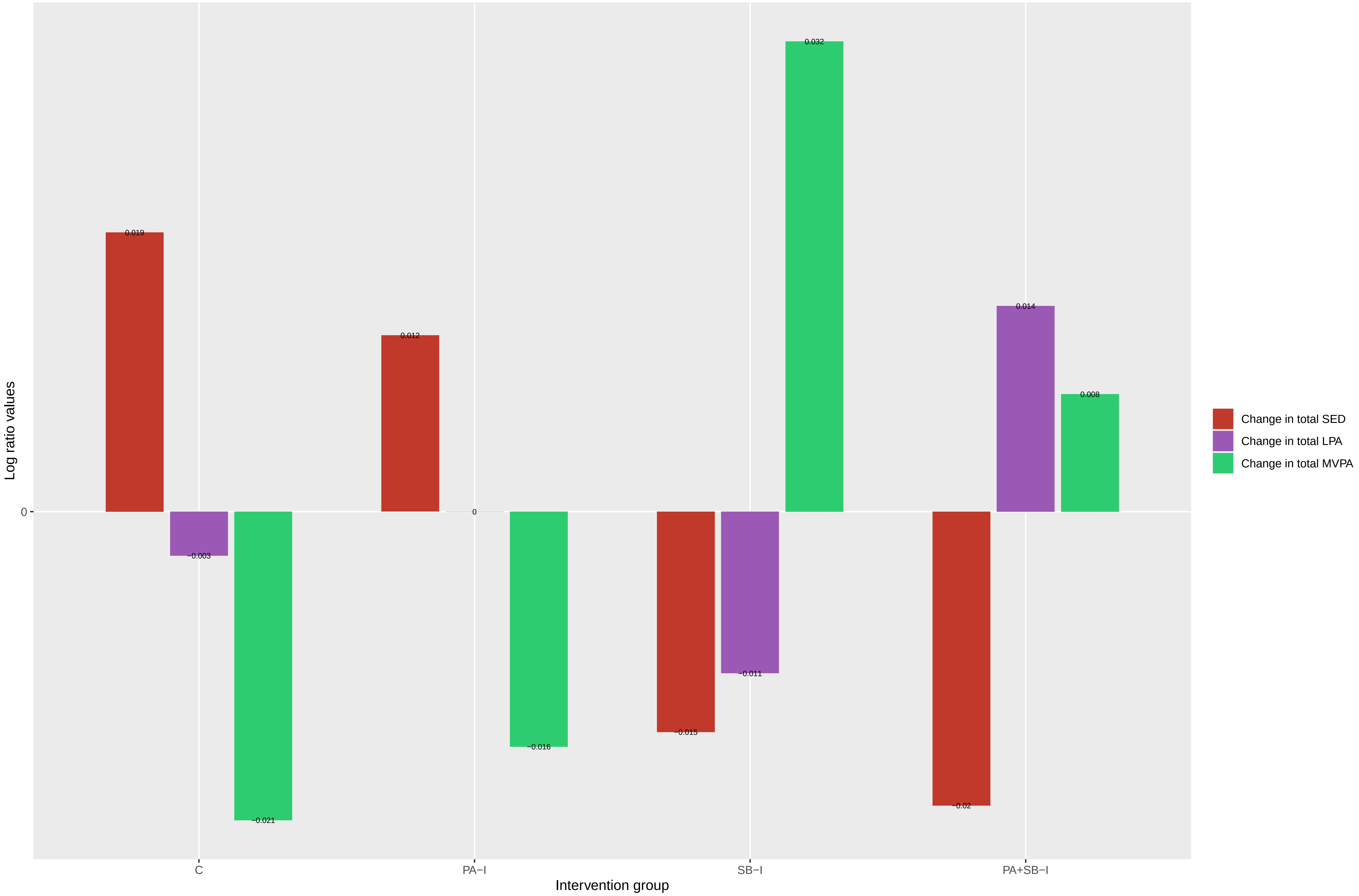

Supplement: Supplementary file 4 — Additional file 4: Figure S2. Baseline movement behaviour accumulation compositions per intervention group. Figure S3. Post-intervention (18 months) movement behaviour accumulation compositions per intervention group. Figure S4. Baseline total volume compositions per intervention group. Figure S5. Post-intervention (18 months) total volume compositions per intervention group. Figure S6. Change between baseline and post-intervention (18 months) in total volume compositions per intervention group. [file 12966_2022_1314_MOESM4_ESM.pdf]
